# Pump the brakes! The hindlimbs of three-toed sloths decelerate and support suspensory locomotion

**DOI:** 10.1242/jeb.245622

**Published:** 2023-04-19

**Authors:** Andrew J. McKamy, Melody W. Young, Angela M. Mossor, Jesse W. Young, Judy A. Avey-Arroyo, Michael C. Granatosky, Michael T. Butcher

**Affiliations:** ^1^ Department of Chemical and Biological Sciences, Youngstown State University, Youngstown, OH 44555, USA; ^2^ Department of Anatomy, New York Institute of Technology College of Osteopathic Medicine, Old Westbury, NY 11568, USA; ^3^ School of Biomedical Sciences, Kent State University, Kent, OH 44243, USA; ^4^ Department of Anatomy and Neurobiology, Northeast Ohio Medical University, Rootstown, OH 44272, USA; ^5^ The Sloth Sanctuary of Costa Rica, Penshurst, Limon 3702, Costa Rica; ^6^ The Dallas World Aquarium, Dallas, TX 75202, USA; ^7^ Center for Biomedical Innovation, New York Institute of Technology College of Osteopathic Medicine, Old Westbury, NY 11568, USA

**Keywords:** Two-toed sloths, Impulse, Inverted quadrupedalism, Kinetics, Stability, *Bradypus variegatus*

## Abstract

Modern tree sloths are one of few mammalian taxa for which quadrupedal suspension is obligatory. Sloth limb musculature is specialized for slow velocity, large force contractions that stabilize their body below branches and conserve energy during locomotion. However, it is unknown whether two- and three-toed sloths converge in their use of limb kinetics and if these patterns are comparable to how primates perform arboreal suspensory locomotion. This study addressed this need by collecting limb loading data in three-toed sloths (*Bradypus variegatus*; *N=*5) during suspensory walking. Sloths performed locomotor trials at their preferred speed on an instrumented beam apparatus with a force platform as the central supporting segment. Peak forces and impulses of the forelimb and hindlimb were recorded and analyzed in three dimensions. The hindlimbs of *B. variegatus* apply large braking forces greater in magnitude than peak forces generated by the forelimbs in propulsion, a pattern consistent with that observed in two-toed sloths. However, *B. variegatus* exhibits hindlimb-biased body weight support in vertical peak forces and impulse, with appreciable laterally directed forces in each limb pair, both of which vary from limb loading distributions in two-toed sloths. Moreover, body weight distribution between limb pairs is opposite to that employed by primates during quadrupedal suspension. Thus, there appear to be multiple strategies for achieving suspensory locomotion in arboreal mammals. These differences may be attributable to anatomical variation or phylogenetic position, but as of yet an explanation remains unknown. Future EMG analyses are expected to provide insight into how specific hindlimb muscle groups contribute to braking forces and stabilizing the center of mass of sloths during suspension.

## Teaser

**Highlighted Article:** Vertical and mediolateral limb loading patterns suggest that there are multiple strategies of body weight support and locomotor stability across suspensory mammals.

## INTRODUCTION

Two-toed sloths (*Choloepus* spp.) and three-toed sloths (*Bradypus* spp.) are rare among mammals in being obligate suspensory taxa (Nyakatura, 2012; Nyakatura and Andrada, 2013; Slater et al., 2016). Moreover, the two modern genera of tree sloths arose from separate lineages within the superorder Xenarthra that split nearly 29 million years ago (Delsuc et al., 2019; Pant et al., 2014), thus the observed similarities in morphology, physiology and lifestyle between *Choloepus* and *Bradypus* represent one of the most remarkable examples of evolutionary convergence (Nyakatura and Fischer, 2011; Pant et al., 2014).

Although *Choloepus* and *Bradypus* generally share patterns of substrate use, species in each genus have their own ecological and behavioral preferences (Adam, 1999; Hayssen, 2009, 2010, 2011), ranging from foraging habits (Montgomery and Sunquist, 1975, 1978) to frequency of suspensory locomotion and posture (Sunquist and Montgomery, 1973; Urbani and Bosque, 2007). Patterns of limb kinematics and kinetics during below-branch locomotion have been previously investigated in *C*. *didactylus* (Granatosky and Schmitt, 2017; Granatosky et al., 2018b; Nyakatura et al., 2010). Similar to suspensory walking (SW) in primates (Granatosky, 2018a; Granatosky and Schmitt, 2017, 2019; Granatosky et al., 2018a), the available evidence reveals that two-toed sloths employ a diagonal-sequence diagonal-couplet (DSDC; Usherwood and Self Davies, 2017) gait, wherein their forelimbs act in net propulsion and their hindlimbs in net braking. These gait characteristics are opposite to the mechanics observed in most upright or pronograde mammals (Granatosky, 2018b; Granatosky et al., 2018a; Gray, 1944; Usherwood and Self Davies, 2017). Two-toed sloths also demonstrate equal body weight support between limb pairs (Granatosky et al., 2018b), with primarily medial forces directed into the substrate by both the forelimbs and hindlimbs to facilitate arboreal stability (Granatosky and Schmitt, 2017). Furthermore, modeling data predict that sloths do not use pendular exchanges of energy during suspensory locomotion (Nyakatura and Andrada, 2013), suggesting that their locomotion is controlled almost entirely by muscle work. Comparatively fewer studies have examined the locomotor features of *Bradypus* (e.g. Gorvet et al., 2020; Mendel, 1985a,b).

Given the degree of morphological convergence among extant species of tree sloths and general physical principles that cannot be overcome, it is reasonable to expect that the locomotor mechanics of *Bradypus* will be similar to those of *Choloepus*. However, while *Choloepus* has limb pairs of nearly equal length (Meritt, 1985; Wislocki, 1928) and feet that are elongate and notably hook shaped (Mendel, 1981a,b), *Bradypus* has relatively long forelimbs but retains much shorter hindlimbs with a long calcaneus and short metatarsals (Marshall et al., 2021). Moreover, *Bradypus* has three partially fused digits on its forefeet compared with two unfused digits (Mendel, 1985a,b) and is smaller in body size than *Choloepus* (Hayssen, 2010, 2011). Such anatomical variations could alter substrate interactions and limb lever mechanics used for suspensory locomotion and posture (Fujiwara et al., 2011; Dickinson et al., 2022). During SW, *Bradypus* flexes its elbow joints, thereby pulling its body towards the substrate to achieve a more level orientation. This position may optimize mechanical advantage of the limb flexor musculature for below-branch stabilization (Olson et al., 2018) and alter patterns of limb loading relative to *Choloepus*. Additionally, *B. variegatus* uses a lateral-sequence diagonal-couplet (LSDC) gait during SW (Gorvet et al., 2020; Mendel, 1985a), perhaps further altering limb loading (Tomita, 1967) versus that of *C. didactylus* with its consistent use of diagonal couplets.

The aim of this study was to evaluate locomotor kinetics in *Bradypus* during SW. It was hypothesized that because of the marked physiological convergence observed between genera, patterns of peak forces and impulse application would generally mimic those observed in *Choloepus*. Specifically, it was predicted that both fore–aft and mediolateral limb loading would be similar in magnitude and directionality between species, but the morphological variation in limb length observed among species would result in differences in vertical body weight support between limb pairs. Moreover, it was expected that magnitudes of propulsive and braking impulses would be equivalently large, signifying how counteracting muscular contraction between forelimb and hindlimb functional groups restrains locomotion in sloths. Such findings could elucidate multiple strategies by which suspensory taxa interact with the substrate to control of inverted quadrupedalism.

## MATERIALS AND METHODS

### Animals and permission

A total of *N=*5 (adult and sub-adult, mean±s.d. body mass: 3.84±0.3 kg) brown-throated three-toed tree sloths (*Bradypus variegatus* Schinz 1825) were used for this study. Sloths were selected for use and handled mainly by staff at the Sloth Sanctuary of Costa Rica (Penshurst, Limon, Costa Rica). All animals were healthy with no visible signs of musculoskeletal or gait abnormalities, and no preference was given to male or female individuals (Tables S1 and S2). Prior to experimentation, sloths were sedated with an injection of Dexdomitor (0.1 ml kg^−1^, injected into the left m. gluteus medius) for percutaneous implantation of fine-wire electrodes into selected hindlimb muscles necessary for simultaneous EMG sampling for a companion study. Animals were weighed on an analog scale to obtain body mass during sedation, which was reversed via injection of Antisedan (0.05 ml kg^−1^), again into the left m. gluteus medius. After the sloths had recovered to full alertness, they were moved to the force beam apparatus for data collection. All experimental procedures complied with the protocols approved by the Costa Rica Ministerio Del Ambiente y Energía, Sistema Nacional de Áreas de Conservación, a través del Programa de Investigación del Área de Conservación La Amistad Caribe (R-SINAC-PNI-ACLAC-012-2021 to M.T.B.).

### Experimental set-up and data collection

**Fig. 1.**
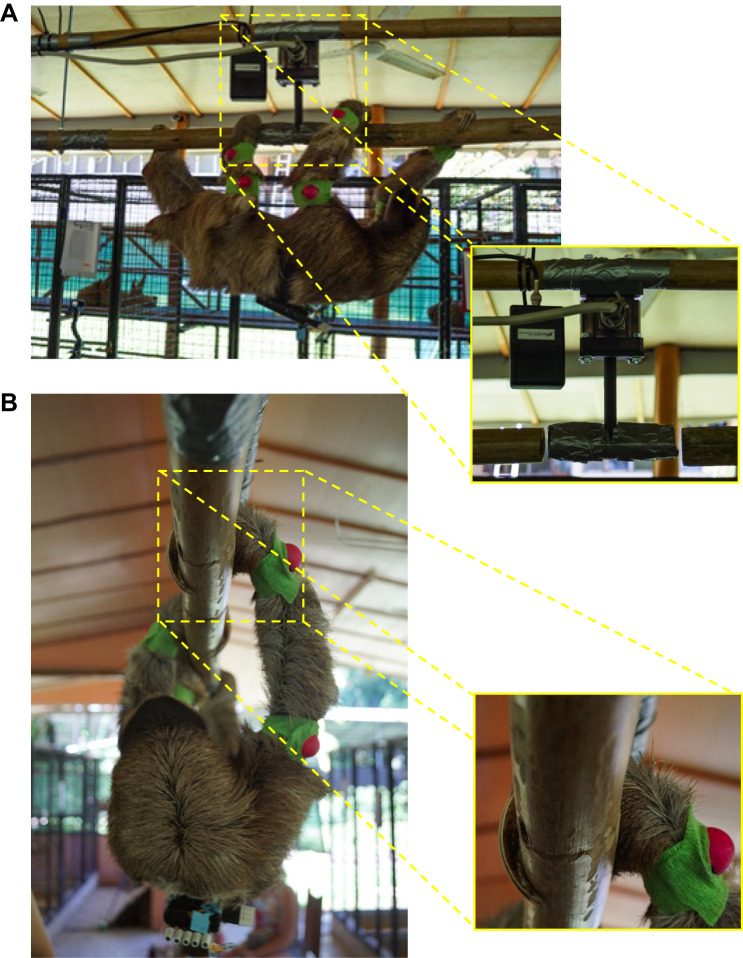
**Force beam apparatus and suspensory walking limb conformation.** (A) Two parallel sections of caña brava wood were secured together with 6.4 mm threaded rods and suspended from the rafters of an overhang with heavy-duty plastic wrap ties. The beam apparatus was reinforced by horizontally oriented heavy-duty plastic wrap ties fastened to animal enclosures. An AMTI force plate (inset) was affixed to the upper beam and bolted to it was a T-shaped grip attachment positioned central to the two lower beam segments. The grip attachment was wrapped in duct tape (and veterinary wrap) to approximate the diameter of caña brava and was positioned with approximately a 5 cm gap between it and either of the two lower beam segments (total length: ∼4.5 m). (B) A three-toed sloth performing suspensory walking. Note the abducted posture of the limbs and placement of the entire palmar/plantar surface of the foot on the dorsal aspect of the beam (inset). Grip on the substrate is anchored by strong flexion of the claws.

Three-dimensional locomotor kinetics were sampled at 1200 Hz using a calibrated, medium-load AMTI load cell (model MC3A, 445 N maximum load; Watertown, MA, USA) placed in between two un-instrumented beams made of caña brava (*Gynerium sagittatum*). The load cell was bolted to a 3D printed, T-shaped grip attachment (i.e. force platform) approximately the same diameter as the caña brava and wrapped with duct tape to provide a frictional pad surface for the animals to grip onto during SW. The end of the T-shaped grip attachment was suspended level between the lower two un-instrumented segments of the beam with ∼5 cm of clearance on either end (Fig. 1A). The animals were allowed to traverse the entire beam in both directions at their preferred speed while being filmed from both sagittal and diagonal views using four GoPro cameras (HERO10, GoPro, San Mateo, CA, USA) set at a frame rate of 60 Hz. Only trials in which the individuals moved in a continuous horizontal path and no visual acceleration or deceleration was observed were considered successful. Among these locomotor trials, only those with clear footfalls on the force platform were saved for subsequent processing and statistical analysis.

Speed calculations were performed following procedures described by Young et al. (2022a). Briefly, 150–200 video frames of a predetermined center of mass (CoM) position (in *C. didactylus*: Granatosky et al., 2018a,b; Nyakatura and Andrada, 2013) from each individual were labeled, in addition to two points of a known distance apart (=106 cm) on the beam apparatus. The position labels were input into markerless pose estimation software (DeepLabCut: Mathis et al., 2018) to train its neural network. The positional outputs as well as the known distance values were used to calibrate the geometric space and provide the conversion factor necessary to calculate the speed within a stride.

### Kinetic analyses

The forces experienced by the force platform were resolved into the vertical (*z*), fore–­aft (*x*) and mediolateral (*y*) components using NetForce software (AMTI, Watertown, MA, USA). In total, forces from *n=*57 forelimb and *n=*90 hindlimb distinct footfalls on the force platform were successfully recorded and analyzed across all five individuals. Review of video records from the locomotor trials, however, revealed bending of the T-shaped grip attachment during limb contact. To compensate for potential errors in force measurement resulting from this compliance, a series of calibrations were made in which the T-shaped grip attachment was loaded with a series of 10 loads (mass range: 0.1–3.40 kg; 0.98–33.4 N). Loading was applied at five locations along the length of the attachment: in the center (directly beneath the load cell) and at distances of 2.5 and 5 cm, both left and right of the center attachment. Calibrations were independently obtained for *z*, *x* and *y* directions. Specifically, a custom-written MATLAB script was used to construct a set of calibration regressions (*R*^2^ for all relationships ≥0.99) that permitted correction for any compliance-related deviations between the applied load and the apparent force registered by the load cell, as well as compensation for cross-talk between orthogonal force channels.

Because of minor technical difficulties with the force platform, it was not possible to estimate potential cross-talk between the *x* and *y* force channels. However, given the low speed of sloth locomotion and small magnitude of fore–aft forces, such electrical cross-talk should have minimal impact on the estimated mediolateral forces. A second custom-written MATLAB script was then used to correct single limb force data from locomotor trials. The script took as input the raw force records and the documented position of the forefoot or hindfoot on the force platform, and returned forces corrected for compliance-related deviations and cross-talk. It is acknowledged that this method does not incorporate any inertial effects of force platform movement itself. However, given the relatively low mass of the T-shaped attachment (140 g), and again the slow locomotor speed of the animals, any inertial effects should be negligible.

Corrected single limb force data (*n=*147 footfalls) were imported into a third custom-written MATLAB script and filtered through a low-pass Fourier filter at 15 Hz to calculate peak forces and impulses from each limb: (1) vertical peak force (V_pk_ force) and impulse (*J*_V_); (2) propulsive peak force (P_pk_ force) and impulse (*J*_P_); (3) braking peak force (B_pk_ force) and impulse (*J*_B_); (4) medial peak force (M_pk_ force) and impulse (*J*_M_); (5) lateral peak force (L_pk_ force) and impulse (*J*_L_). All recorded kinetics data were also corrected for direction of travel, orientation and whether the contact limb was right or left. Specifically, fore–aft forces applied by the animal were split into negative braking and positive propulsive forces, and mediolateral forces applied by the animal were segregated such that medially directed forces were negative and laterally directed forces were positive. Additionally, the total area under the horizontal component of the force–time curve was measured to determine the net fore–aft impulse. The latter provides a means for differentiating the overall functional role of a limb during locomotor behavior (Granatosky et al., 2018a) such that positive (+) values indicate a net propulsive limb, whereas negative (−) values indicate a net braking limb. Values approximating zero represent single limb forces, wherein braking and propulsive impulses are equal. The raw force data and MATLAB analysis scripts are available from M.C.G. upon reasonable request.

Peak force (in N) and impulse (in N s) in each direction were normalized to percentage body weight (% BW) and percentage body weight seconds (% BWS), respectively, to allow for normalized comparison between individuals (Granatosky and Schmitt, 2017). All values were averaged across individuals and reported as pooled means±s.d. Descriptive statistics for each individual sampled are presented in Tables S1–S4. Last, a separate evaluation of body weight support via impulse data was limited to a subset of trials (*n=*20) for which there were consecutive forelimb and hindlimb contacts (i.e. within a single stride) on the force platform. For this analysis, values of *J*_V_, *J*_P_ and *J*_B_ were used to calculate percentages of total impulse to assess the relative contribution to body weight support by each limb pair.

### Statistics

Statistical tests were conducted using R (http://www.R-project.org/). The R packages ‘nlme’ (http://CRAN.R-project.org/package=nlme) and ‘emmeans’ (Pinheiro and Bates, 2000) were used for these analyses. For the purposes of statistical testing, the absolute values of all recorded forces were used, as the goal was to compare magnitudes, rather than direction per se. To assess normality and homoscedasticity in the datasets, Shapiro–Wilk and Levene's tests, respectively, were conducted and peak force, impulse and speed were rank-transformed prior to conducting statistical testing (Sokal and Rohlf, 2012). A series of full-factorial linear mixed effect (LME) models were created to assess differences in vertical, fore–aft and mediolateral peak forces and impulses between limb pairs (i.e. intraspecific variation) and between species (i.e. interspecific variation) by using available data from *C. didactylus* (Granatosky and Schmitt, 2017; Granatosky et al., 2018b). As speed is known to influence patterns of limb loading (Granatosky et al., 2020), it was maintained as a fixed covariate in each model tested. Individual idiosyncrasies were also accounted for by using individual as a random effect in each model (Bates et al., 2015; Winter, 2013 preprint). Models were specified with main effects for limb, species and speed, a factor-by-factor interaction between species and limb, and (where significant) factor-by-covariate interactions between species and speed.

## RESULTS

**Fig. 2.**
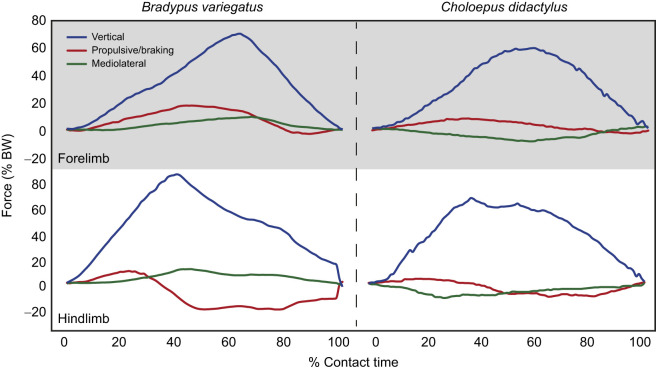
**Representative single limb forces sampled from *Bradypus variegatus* and *Choloepus didactylus* during suspensory walking.** Vertical (blue, *z*-axis), fore–aft (red, *x*-axis) and mediolateral (green, *y*-axis) forces applied by the forelimbs (above, shaded) and hindlimbs (below, not shaded). Vertical forces are shown as positive values by convention, as well as positive propulsive force and negative braking force. Medially directed forces are negative and laterally directed forces are positive. All values were normalized to percentage body weight (% BW) and the data shown are from consecutive limb contacts within the same trial. Data for *C*. *didactylus* are from Granatosky and Schmitt (2017) and Granatosky et al. (2018b).

**Fig. 3.**
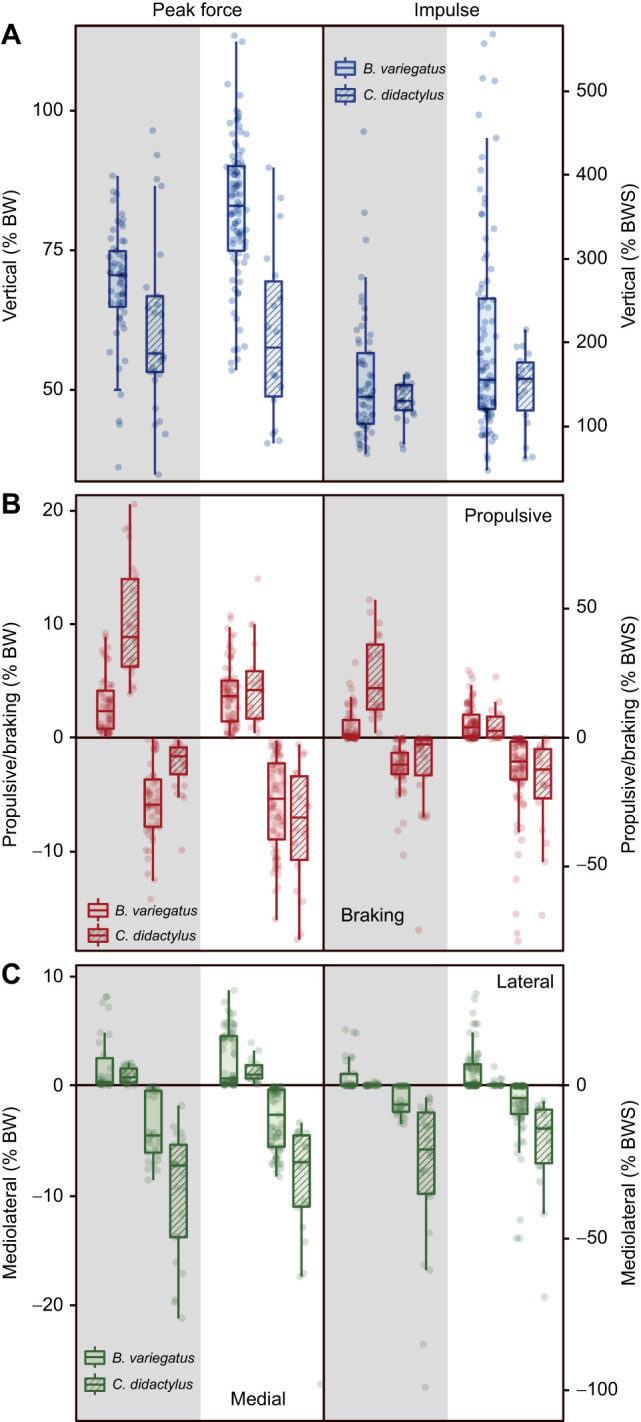
**Box and whisker plots of relative peak forces and impulses between limb pairs and species during suspensory walking.** (A) Comparisons of vertical peak forces (left) and impulses (right) between forelimbs (shaded) and hindlimbs (not shaded) for both species. (B) Comparisons of peak (+) propulsive versus (−) braking forces (left) and impulses (right) between forelimbs (shaded) and hindlimbs (not shaded) for both species. (C) Comparisons of peak (−) medial versus (+) lateral forces (left) and impulses (right) between forelimbs (shaded) and hindlimbs (not shaded) for both species. Color coding is the same as in Fig. 2. All values of force were normalized to percentage body weight (% BW) and those for impulse to percentage body weight seconds (% BWS). Box plots show median values, upper and lower quartiles and 1.5× interquartile range data from *B. variegatus* (*N*=5) and *C. didactylus* (*N*=2; Granatosky and Schmitt, 2017; Granatosky et al., 2018b). Given that speed was also entered as a covariate in the LME models, these box plots are intended to represent the spread of the data across limb pairs and species, rather than depict the actual statistical test results used for analyses.

The overall patterns of limb loading for each limb pair of *B. variegatus* are shown in Fig. 2 and mean values of peak force and impulse in each direction are presented in Tables 1 and 2, respectively. After accounting for speed variation and individual differences, the LME model results demonstrate that the hindlimbs of *B. variegatus* apply larger V_pk_ forces (*P*<0.001) than the forelimbs (82.0±13.2% BW versus 68.6±10.6% BW, respectively) (Tables 1 and 3, Fig. 3A), as well as larger magnitudes of *J*_V_ (*P*=0.004), but neither is significantly different between the limb pairs of *C. didactylus* (both *P*≥0.135: Table 3; Table S3). Greater P_pk_ forces (*P*<0.001) are exerted by the forelimbs of *B. variegatus*, whereas greater B_pk_ forces (*P*<0.001) are applied by the hindlimbs (Tables 1 and 3, Fig. 3B), making forelimbs net propulsive (23.6% BWS) and hindlimbs net braking (−40.7% BWS) during SW (Table 2). This intraspecific trend is consistent with that of *C. didactylus*, wherein only P_pk_ and B_pk_ force magnitudes vary significantly between limb pairs (both *P*≤0.009: Table 3). Collectively, the findings for fore–aft peak forces parallel LME model results for *J*_P_ and *J*_B_ in each limb pair within both species (all *P*<0.001; Table S3), although peak *J*_B_ does not vary significantly (*P*=0.063) between the limb pairs of *C. didactylus*. The hindlimbs of *B. variegatus* also apply larger M_pk_ forces (*P*<0.001) and *J*_M_ (*P*=0.001) than the forelimbs, but the two limb pairs exert similarly large L_pk_ forces and *J*_L_ (both *P*≥0.347: Tables 2 and 3; Table S3). The means of L_pk_ forces, however, are appreciable for the forelimbs and hindlimbs of *B. variegatus* (6.5±5.7% BW versus 6.1±7.5% BW, respectively) (Table 1, Fig. 3C). In general, mediolateral peak forces and impulses are not statistically different between limb pairs of *C. didactylus* (all *P*≥0.356: Table 3; Table S3).

**Table 1.**
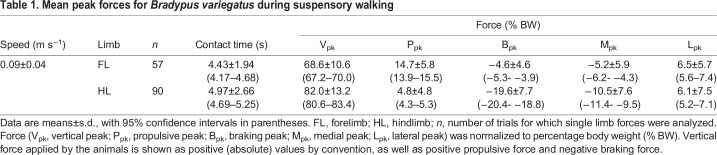
Mean peak forces for *Bradypus variegatus* during suspensory walking

**Table 2.**
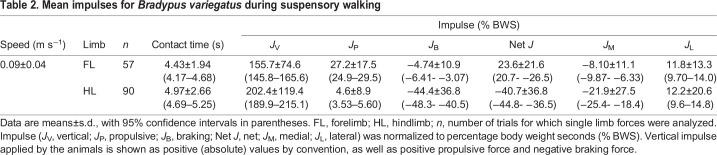
Mean impulses for *Bradypus variegatus* during suspensory walking

**Table 3.**
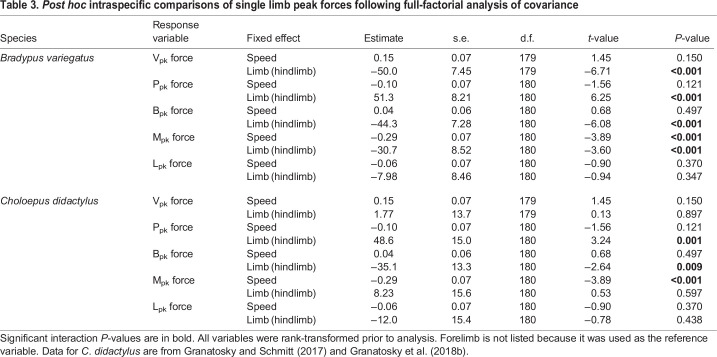
*Post hoc* intraspecific comparisons of single limb peak forces following full-factorial analysis of covariance

**Fig. 4.**
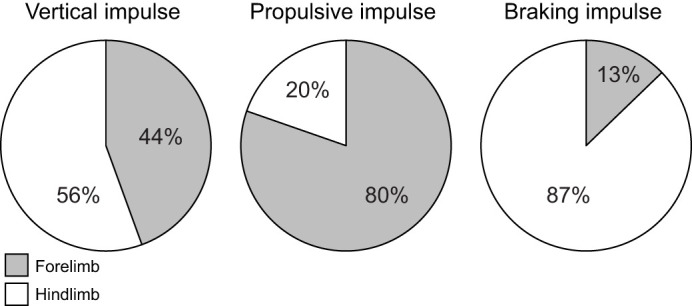
**Relative contribution of forelimbs and hindlimbs to body weight support in *B. variegatus* during suspensory walking.** Percentage of vertical, propulsive and braking impulses (single limb impulse/total impulse) applied by the forelimbs (shaded) and hindlimbs (not shaded). Values of relative impulse were determined as the quotient of impulse in each direction per limb and total vertical impulse across limb pairs during a single stride. Data shown represent a subset of *n=*20 strides.

Interspecific differences are largely reflective of the variation in peak loading between limb pairs within species. In particular, V_pk_ forces are significantly greater in the forelimbs (*P*=0.013) and hindlimbs (*P*≤0.001) of *B. variegatus* versus *C. didactylus*, although forelimb V_pk_ forces are only significant at the maximum range, while hindlimb V_pk_ forces are significant at both the mean and maximum values of the overlapping speed range (Table 4). Whereas magnitudes of *J*_V_ observed between limb pairs and species are not significantly different (all *P*>0.135; Table S4), the significantly larger mean values of *J*_V_ for the hindlimbs of *B. variegatus* (Table 2; Tables S3 and S5) indicate a greater percentage of vertical body weight support relative to the forelimbs in three-toed sloths (Fig. 4). In addition, B_pk_ forces exerted by each limb pair are larger in *B. variegatus* than in *C. didactylus* (both *P*≤0.016: Table 4), which corresponds with greater net (–) fore–aft impulse (*P*=0.024) applied in braking by the hindlimbs of *B. variegatus* (Table S4). However, there are no significant differences in either peak *J*_P_ (*P*≥0.249) or *J*_B_ (*P*≥0.073) generated by the forelimbs and hindlimbs between species (Table S4). Despite comparable M_pk_ forces and *J*_M_ applied by the limb pairs of both species (all *P*≥0.261: Table 4; Table S4), the means of L_pk_ forces and *J*_L_ for the forelimbs (both *P*≤0.007) and hindlimbs (both *P*≤0.013) in *B. variegatus* are significantly larger than those in *C. didactylus* (Table 4; Table S4).

**Table 4.**
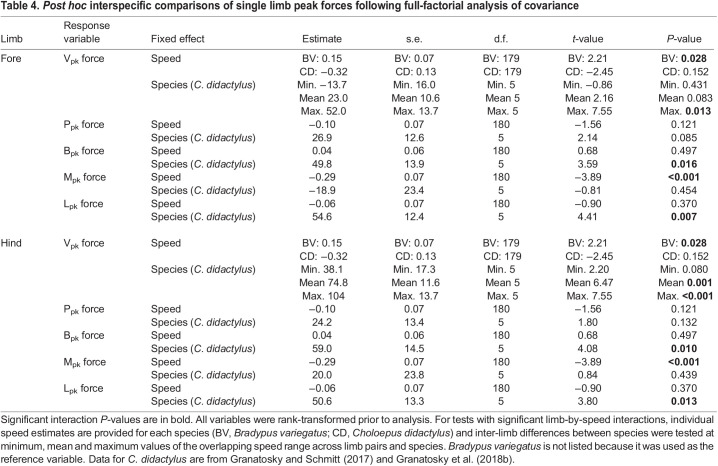
*Post hoc* interspecific comparisons of single limb peak forces following full-factorial analysis of covariance

## DISCUSSION

These novel findings along with data from recent studies (Granatosky and Schmitt, 2017; Young et al., 2023) on suspensory locomotor kinetics in tree sloths reveal three major findings. First, as for *C. didactylus*, there is a functional difference between the limb pairs of *B. variegatus*, with the forelimbs and the hindlimbs serving as the major propulsive and braking appendages, respectively. Second, although body weight and impulse are equally distributed between limb pairs during SW in *C. didactylus* (Granatosky et al., 2018b), this feature may be exclusive to two-toed sloths (Dickinson et al., 2022) because *B. variegatus* demonstrates a hindlimb bias in vertical body weight support. Third, *C. didactylus* mainly exerts medially directed forces with both limb pairs (Granatosky and Schmitt, 2017), whereas medial peak force predominates in the hindlimbs of *B. variegatus* and, surprisingly, both of its limb pairs exert significant magnitudes of laterally directed forces. This latter finding is novel, as neither two-toed sloths nor suspensory primates apply appreciable magnitudes of lateral peak force during inverted quadrupedalism.

Limb loading patterns resolved for SW in both genera indicate that the hindlimbs act as the main braking and stabilizing limbs during SW as originally hypothesized by Goffart (1971), a convergent trait among tree sloths (Granatosky and Schmitt, 2017; Nyakatura and Andrada, 2013), bats (Granatosky, 2018a) and numerous primates (e.g. lemurs and monkeys: Dickinson et al., 2022; Granatosky et al., 2016; Granatosky and Schmitt, 2019). Thus, in addition to brachiation, use of the forelimbs to provide primary propulsion for inverted quadrupedalism suggests this to be a universal locomotor strategy for suspensory taxa. Sloths also employ slow, intermittent locomotion as an adaptive strategy to conserve metabolic energy (Gorvet et al., 2020; McDonald and De Iuliis, 2008) and this movement pattern matches well with a broad distribution of slow-contracting muscle fibers dependent on anaerobic metabolism (Spainhower et al., 2018, 2021). For example, the hindlimbs of *B. variegatus* contain several muscles that are homogeneous in their expression of slow-contracting MHC-1 fibers (Spainhower et al., 2021) and these may be the most extremely well suited for applying braking and stabilizing forces.

Animals in this study were noted to reach with their forelimbs ahead of their body position to test the beam for adequate strength/stiffness prior to committing to support of their body weight. It is not known, however, whether *Choloepus* is as deliberate with their purchase of the substrate at touchdown as *Bradypus*. It was additionally observed that compared with *C. didactylus* during the contact phase of SW, the hindlimb of *B. variegatus* initially provides similarly modest propulsive impulse, and transitions early rather than later in support to applying significantly larger braking force/impulse. Conversely, its forelimbs provide large propulsive force/impulse and transition late in contact phase to exert equally modest braking force prior to lift-off (Fig. 2). Because these patterns of fore–aft limb loading are generally similar to those observed in *Choloepus* (Granatosky and Schmitt, 2017), this may be the mechanism by which sloths minimize swinging in the sagittal plane at the beginning and end of the contact phase during SW and precisely control their forward horizontal speed. As previously posited by Nyakatura and Andrada (2013), sloth locomotion may be entirely driven by muscle work and the transitions of propulsive to braking forces shown between limb pairs likely reflect this type of limb muscle function. Previous work in *B. variegatus* showed the possibility for co-activation of selected flexor/adductor muscles in each limb pair (Gorvet et al., 2020), which may ensure that there is minimal horizontal acceleration of the CoM via balancing of the propulsive and braking forces, as was suggested for inverted quadrupedalism in slow lorises (*Nycticebus*; Ishida et al., 1990). In sloths, large but very slow-contracting motor units can be selectively recruited (Gorvet et al., 2020) to provide equal propulsion and braking across a stride, thus producing controlled movements that reduce oscillations of the substrate and minimize energy loss.

A divergent pattern between genera of tree sloths is related to limb proportions. Three-toed sloths have elongate forelimbs relative to both their body and hindlimb lengths, a morphological trait that they share with suspensory primates (Granatosky, 2018c), and one that also might be related to vertical climbing performance on larger diameter substrates (Jungers, 1978). For example, *B. variegatus* has a higher intermembral index (IMI: 1.65±0.11 versus 1.11±0.03) and ankle extensor index (AEI: 0.73±0.04 versus 0.63±0.07) relative to those of *C. didactylus* (Marshall et al., 2021). Greater trochanter height index and crural index, however, are both lower in *B. variegatus*, jointly suggesting that the hindlimbs of three-toed sloths have enhanced limb mechanical advantage (MA) and large out-force application to the substrate during locomotion and posture (Marshall et al., 2021), consistent with a greater role in support of the body weight in suspension. Retention of shorter hindlimbs could have contributed to convergence of support postures (e.g. tripodal posture) in suspensory mammals with a high IMI. Nevertheless, having greatly elongate forelimbs may limit the options in which *Bradypus* achieves support via vertical limb loading, and although their forelimb bones are stronger (compressive and bending strength) than their hindlimb bones (Mossor et al., 2022), both limb pairs are capable of resisting routine tensile loading and this capacity is equivalent between two- and three-toed forms. Therefore, large MA of the well-developed flexor musculature in *B. variegatus* (Mendel, 1985a; Morgan et al., 2022) by modified origins and insertions (Butcher et al., 2022) could be the most critical factor for a greater reliance on the hindlimbs for vertical body weight support.

Primates exhibit hindlimb-biased support during pronograde arboreal locomotion accounting for 55–70% of the body weight (Demes et al., 1994; Larson and Demes, 2011). These magnitudes match patterns of hindlimb loading during SW in *B. variegatus* (Table 1). Yet, the available data for primates based on vertical peak force and impulse (Ishida et al., 1990; Granatosky et al., 2016; Dickinson et al., 2022) indicate a shift to forelimb-biased support during inverted quadrupedalism (Granatosky and Schmitt, 2019). Indeed, a hindlimb-biased support distribution is rare among inverted quadrupeds, with the giant flying fox (*Pteropus vampyru*s) being the only other species besides *B. variegatus* to exhibit such a pattern, and the mechanisms for support vary considerably among suspensorial species. For two-toed sloths, the position of the CoM and limb kinematics relative to the CoM provide a good explanation for body weight support distribution equally between the limbs. In primates, however, the mechanism is less well known, and it remains unclear whether CoM position or relative contact duration between the limbs determines patterns of forelimb versus hindlimb body weight distribution (Dickinson et al., 2022). More explicit tests of CoM position during suspensory locomotion are required to determine the mechanism and ecological advantage of hindlimb-biased support in *B. variegatus.*

Though the magnitudes of vertical peak force in the hindlimbs of *B. variegatus* are significantly larger compared with those in their forelimbs, as well as those from the hindlimbs of *C. didactylus* (Tables 3 and 4), the vertical impulses evaluated across a stride provide the most direct evidence of hindlimb-biased support in three-toed sloths (Fig. 4). The suggestion of hindlimb-biased suspensory support in *Bradypus*, however, could be a consequence of selection for climbing ability. For example, appreciable MA at the hip and knee joints versus high velocity of joint rotation, but especially large MA at the ankle joint (Marshall et al., 2021) is beneficial for slow, stealthy climbing behavior that involves prolonged vertical clinging. Gripping the substrate via low velocity, high force contractions of distal hindlimb musculature (Spainhower et al., 2021) would also provide stability in both vertical and horizontal directions.

Another divergent pattern between genera of tree sloths is reflected in the magnitudes of both peak braking and medial forces exerted on the substrate by the hindlimbs of *B. variegatus* that are nearly double those of *C. didactylus*. In particular, significantly greater braking forces may prevent cranial shifting of position of the CoM and body weight onto the forelimb (i.e. horizontal levering: Granatosky et al., 2018b), especially in three-toed sloths that move at consistently slower average velocities (Britton, 1941; Gorvet et al., 2020). The m. sartorius (hip/knee flexor) and m. iliopsoas (hip flexor), as well as the bellies of m. adductor longus, were formerly hypothesized as the muscles with potentially the greatest capacity for applying braking and medial forces, respectively (Spainhower et al., 2021; Butcher et al., 2022). It is also possible that the m. quadriceps (knee extensor) in *B. variegatus* enhance the strut-like function of the hindlimb by undergoing isometric contraction (i.e. adding to the net braking forces), in addition to a role in stabilizing the knee joint by counteracting large flexor torques applied by the massive, forceful knee flexor musculature (Butcher et al., 2022). However, muscle fiber architecture (Morgan et al., 2022) and EMG activation analyses in the hindlimb of three-toed sloths are needed to verify such roles of these functional muscle groups.

The finding of significantly elevated lateral peak forces and impulse by the forelimbs and hindlimbs of *Bradypus* (7.2–8.5% BW), as opposed to minimal lateral peak forces in *Choloepus* (1.0–1.4% BW), is another distinction in the way that the feet of tree sloths interact with the substrate. Initial purchase of the substrate varies between species, with *B. variegatus* placing the entire plantar/palmar aspect of its feet in contact with the upper surface of the substrate, with flexion of the claws to anchor the limb (Gorvet et al., 2020; Mendel, 1985a) (see Fig. 1B), whereas *C. didactylus* prefers contacting the substrate with only its claws (Granatosky et al., 2018b; Mendel, 1981b). The magnitudes of peak laterally directed forces observed could be caused by the claws first pulling laterally against the substrate to secure the purchase just before the entire foot contours to the substrate and full weight is supported by the limb. Some amount of the lateral peak forces observed may additionally represent simultaneous abduction and or lateral rotation as the limbs are retracted during intervals of propulsion (Fig. 1B), whereas medially directed forces are those that stabilize the body by contralateral limbs as previously found in *C. didactylus* (Granatosky and Schmitt, 2017).

The limb placement of the LSDC gait in *B. variegatus* (Gorvet et al., 2020) also serves to diagonally balance sloths during suspensory locomotion, such that the detached and protracted forelimb is afforded the freedom to test for purchase of new substrates, in addition to allowing for a large range of motion needed in a complex arboreal environment. As *Bradypus* prefers the high-canopy and/or emergent level of the rainforest, and they must navigate a more vertical strata niche than *Choloepus* (Hayssen, 2010, 2011), a greater range of motion for the forelimbs may be more critical to their locomotor success. During functional use of tripodal support, both hindlimbs have the role of anchoring the animal to the substrate, which is realized by their large grip forces that approximate or slightly exceed body weight force, especially on larger diameter substrates (Young et al., 2022b). Moreover, this posture mirrors that of hang-feeding behavior in orangutans (Hunt, 2016) and spider monkeys (Youlatos, 2002), which support their body weight by grasping the substrate with their hindlimbs and prehensile tail (i.e. tail-assisted hindlimb hanging) to free the forelimbs for foraging on fruit.

### Limitations

Despite the adequate sample size and large number of single limb contacts on the force platform, there are still several possible limitations to this study. One such consideration is that the animals in this study were not always undergoing steady-state locomotion. Those trials were excluded from analysis and were mainly from one individual (Bv2) for which we had a disproportionately large number of recorded footfalls (see Tables S1 and S2). That said, data for this individual still represent the highest measured velocities in the dataset. Minor accelerations and decelerations, however, are typical of intermittent sloth locomotion. Another potential limitation is the sedation effects of the drug Dexdomitor injected prior to experimentation, which was necessary for implantation of fine wire EMG electrodes used in our companion study and the benefit of collecting simultaneous *in vivo* data. This sedative, though countered with a reversal agent (Antisedan) prior to trials of SW, may have initially reduced performance if the individuals were not able to fully metabolize the drug within the time given to recover. Nevertheless, these two drugs were used in a prior EMG study (Gorvet et al., 2020) with no obvious performance impairment. Furthermore, the sloths used in our study are routinely exposed to sedation/reversal for health checks, claw trimming and various veterinary medical procedures. A final possible limitation involves the construction of the force beam apparatus. Although composed of a local woody grass (caña brava), the beam is not typical of the substrate (i.e. *Cecropia* trees) that *Bradypus* uses. The small diameter and smooth surface of the beam were therefore unnatural and may have altered how the sloths gripped and walked (or were motivated to walk) across the substrate.

### Conclusions

Despite their numerous shared physiological traits, suspension in sloths exemplifies a combination of convergent and divergent locomotor kinetics. Consistent between genera, and suspensory primates, is the use of the forelimbs for propulsion and the hindlimbs for both braking and stability. However, appreciable magnitudes of both medial and lateral peak forces and impulses exerted by each limb pair and being hindlimb biased in body weight support signify limb loading patterns in *Bradypus* that diverge not only from those in *Choloepus* but also from those in primates. Thus, inverted quadrupedalism involves a continuum of mechanics that are employed across suspensorial taxa, which is dependent on several factors, including limb proportions, bone strength, modifications to the flexor musculature for enhanced mechanical advantage, behavioral flexibility and ecological preferences. In addition, an overriding consideration for tree sloths is slow, deliberate substrate use that ensures stability realized when testing and traversing arboreal supports.

## Supplementary Material

10.1242/jexbio.245622_sup1 Supplementary information
