# Supplementary material for: Pump the brakes! The hindlimbs of three-toed sloths decelerate and support suspensory locomotion
Source: J Exp Biol. 2023 Apr 19;226(8):jeb245622. doi: 10.1242/jeb.245622 (PMC10263148; doi:10.1242/jeb.245622)
Supplement: Supplementary information [file jexbio-226-245622-s1.pdf]

Table S1. Means (±s.d.) of peak forces (normalized in %BW) for *N*=5 individuals of *Bradypus variegatus*.

	Body Mass (kg)	Sex	Speed (ms^{-1})	Limb	*N*	Contact Time (s)	V_{pk} (%BW)	P_{pk} (%BW)	B_{pk} (%BW)	$M_{p}k$ (%BW)	L_{pk} (%BW)
BV1	3.4	M	0.08±0.03	FL	16	4.45±1.5	68.3±8.5	14.2±5.5	-6.5±5.1	-4.6±6.2	8.1±6.5
						(4.07-4.82)	(66.2-70.3)	(12.8-15.5)	(-7.9-5.2)	(-7.9-5.2)	(6.0-10.1)
				HL	20	4.49±1.95	73.5±12.1	8.4±7.2	-14.5±6.7	-9.4±6.9	3.6±5.1
						(4.06-4.93)	(70.8-76.2)	(6.7-10.0)	(-16.0-13.0)	(-11.3-7.6)	(2.3-5.0)
BV2	4.0	M	0.11±0.04	FL	19	3.26±1.22	64.4±8.3	13.4±5.8	-4.4±4.6	-4.6±5.9	6.4±5.2
						(2.98-3.54)	(62.5-66.4)	(12.1-14.7)	(-5.8-3.0)	(-6.1-3.1)	(5.1-7.8)
				HL	43	4.27±2.22	86.2±12.7	4.2±4.3	-21.5±7.2	-10.9±7.7	7.1±8.7
						(3.93-4.61)	(84.3-88.2)	(3.6-4.9)	(-22.6-20.4)	(-12.4-9.5)	(5.5-8.8)
BV3	4.2	M	0.04±0.02	FL	1	9.40	76.6	12.9	--	-0.70	11.3
				HL	4	9.64±2.92	83.3±5.2	3.5	-22.5±6.9	-13.5±9.2	4.7±8.2
						(8.18-11.11)	(80.7-85.9)	--	(-26.0-19.1)	(-18.1-9.0)	(0.62-8.8)
BV4	3.9	F	0.08±0.02	FL	14	4.49±1.10	71.6±8.0	17.6±5.5	-3.1±4.0	-4.9±5.9	6.8±6.9
						(4.20-4.79)	(69.5-73.7)	(16.2-19.1)	(-4.2-1.9)	(-7.3-2.4)	(4.0-9.6)
				HL	16	4.15±0.90	78.3±15.9	4.1±4.3	-18.4±8.5	-12.1±7.9	4.2±4.7
						(3.92-4.37)	(74.3-82.2)	(2.9-5.3)	(-20.5-16.3)	(-14.7-9.4)	(2.6-5.8)
BV5	3.7	M	0.07±0.02	FL	5	7.99±2.19	55.6±15.2	10.0±1.9	-2.6±3.5	-6.1±5.7	4.4±5.4
						(7.01-8.97)	(48.8-62.3)	(9.2-10.9)	(-4.6-0.6)	(-8.6-3.6)	(2.0-6.8)
				HL	9	8.46±3.20	82.8±6.3	4.8±3.2	-19.0±10.4	-8.5±7.5	8.5±8.0
						(7.40-9.53)	(80.7-84.9)	(3.3-6.2)	(-22.4-15.5)	(-11.0-6.0)	(5.8-11.1)

N, number of single limb forces analyzed; FL, forelimb; HL, hindlimb; BW, bodyweight; V_{pk}, vertical peak force; P_{pk}, propulsive peak force; B_{pk}, braking peak force; M_{pk}, medial peak force; L_{pk}, lateral peak force.

In parentheses are 95% confidence intervals.

Table S2. Means (±s.d.) of impulses (normalized in %BWS) for *N*=5 individuals of *Bradypus variegatus*.

	Body Mass (kg)	Sex	Speed (ms^{-1})	Limb	*N*	Contact Time (s)	J_V (%BWS)	J_P (%BWS)	J_B (%BWS)	J_M (%BWS)	J_L (%BWS)
BV1	3.4	M	0.08±0.03	FL	16	4.45±1.5	183.1±92.2	24.0±12.8	-8.1±16.4	-6.6±10.9	8.1±6.5
						(4.07-4.82)	(160.1-206.2)	(20.8-27.2)	(-12.3-3.8)	(-10.0-3.1)	(6.0-10.1)
				HL	20	4.49±1.95	182.6±120	10.2±13.9	-24.4±14.6	-15.0±11.0	5.0±9.8
						(4.06-4.93)	(155.8-209.4)	(7.0-13.4)	(-27.7-21.1)	(-18.1-12.0)	(2.4-7.6)
BV2	4.0	M	0.11±0.04	FL	19	3.26±1.22	101.6±29.9	18.4±17.4	-2.6±4.9	-4.58±5.9	8.0±6.8
						(2.98-3.54)	(94.8-108.5)	(14.4-22.4)	(-4.0-1.1)	(-6.1-3.1)	(6.2-9.8)
				HL	43	4.27±2.22	182.0±106.1	3.1±7.6	-21.5±7.2	-17.5±24.6	11.8±19.3
						(3.93-4.61)	(165.8-198.2)	(1.9-4.3)	(-22.6-20.4)	(-22.2-12.9)	(8.1-15.4)
BV3	4.2	M	0.03±0.02	FL	1	9.40	355.4	60.1	--	-0.28	40.8
				HL	4	9.64±2.92	414.1±125.6	3.44	-129.0±69.0	-71.6±54.8	14.6±28.9
						(8.18-11.11)	(351.3-476.9)	--	(-163.5-94.5)	(-98.9-44.2)	(0.17-29.1)
BV4	3.9	F	0.08±0.02	FL	14	4.49±1.10	160.6±35.1	35.6±15.6	-3.1±4.0	-7.7±12.0	11.7±12.6
						(4.20-4.79)	(151.2-170.0)	(31.4-40.0)	(-4.2-1.9)	(-12.6-2.8)	(6.5-16.8)
				HL	16	4.15±0.90	143.5±49.1	3.6±6.1	-33.8±16.7	-16.9±11.3	5.1±8.0
						(3.92-4.37)	(131.3-155.8)	(1.8-5.4)	(-38.0-29.6)	(-20.6-13.1)	(2.4-7.8)
BV5	3.7	M	0.07±0.02	FL	5	7.99±2.19	240.4±27.3	38.0±21.2	-1.3±1.9	-16.5±15.5	17.0±24.6
						(7.01-8.97)	(228.2-252.6)	(28.5-47.4)	(-2.45-0.22)	(-23.5-9.6)	(6.0-28.0)
				HL	9	8.46±3.20	333.4±75.3	8.6±11.7	-65.9±43.4	-28.3±27.3	28.4±32.3
						(7.40-9.53)	(308.3-358.5)	(3.4-13.8)	(-80.4-51.5)	(-37.4-19.2)	(17.6-39.1)

N, number of trials for which consecutive fore- and hindlimb forces were collected; FL, forelimb; HL, hindlimb; BWS, bodyweight seconds; J_V, vertical impulse; J_P, propulsive impulse; J_B, braking impulse; J_M, medial impulse; J_L, lateral impulse.

In parentheses are 95% confidence intervals, except for duty factor, which represents the range of measurements.

Table S3. Post hoc *intraspecific* comparisons of single limb impulses following full-factorial analysis of covariance.

Species	Response Variable	Fixed Effect	Estimate	Standard Error	df	t-value	*p*-value
Bradypus variegatus	Peak Vertical Impulse	Speed	-0.371	0.0772	179	-4.81	**<0.001**
		Limb (hindlimb)	-22.7	14.15	179	-2.93	**0.004**
	Peak Propulsive Impulse	Speed	-0.159	0.0666	180	-2.39	**0.018**
		Limb (hindlimb)	50.2	7.79	180	6.45	**<0.001**
	Peak Braking Impulse	Speed	-0.152	0.0675	180	-2.26	**0.025**
		Limb (hindlimb)	-46.6	7.92	180	-5.88	**<0.001**
	Peak Medial Impulse	Speed	-0.344	0.0730	180	-4.71	**<0.001**
		Limb (hindlimb)	-28.3	8.64	180	-3.30	**0.001**
	Peak Lateral Impulse	Speed	-0.152	0.0649	180	-3.28	**0.020**
		Limb (hindlimb)	-1.98	8.19	180	-0.241	0.810
	Peak *Net* Fore-Aft Impulse	Speed	FL: -0.133<br>HL:0.148	FL: 0.063<br>HL: 0.058	FL: 179<br>HL:179	FL: -2.10<br>HL:2.55	FL: **0.037**<br>HL: **0.012**
		Limb (hindlimb)	Min: 114.9<br>Mean: 92.9<br>Max: 75.4	Min: 7.74<br>Mean: 5.38<br>Max: 8.14	Min: 179<br>Mean:179<br>Max: 179	Min: 14.8<br>Mean: 17.3<br>Max: 9.27	Min: **<0.001**<br>Mean: **<0.001**<br>Max: **<0.001**
Choloepus didactylus	Peak Vertical Impulse	Speed	-0.371	0.0772	179	-4.81	**<0.001**
		Limb (hindlimb)	-21.3	14.15	179	-1.50	0.135
	Peak Propulsive Impulse	Speed	-0.159	0.0666	180	-2.39	**0.018**
		Limb (hindlimb)	63.3	14.2	180	4.46	**<0.001**
	Peak Braking Impulse	Speed	-0.152	0.0675	180	-2.26	**0.025**
		Limb (hindlimb)	-27.0	14.445	180	-1.87	0.063
	Peak Medial Impulse	Speed	-0.344	0.0730	180	-4.71	**<0.001**
		Limb (hindlimb)	8.13	15.8	180	0.516	0.606
	Peak Lateral Impulse	Speed	-0.344	0.0730	180	-4.71	**<0.001**
		Limb (hindlimb)	-13.6	14.96	180	-0.909	0.365

Journal of Experimental Biology • Supplementary information

	Peak *Net* Fore-Aft Impulse	Speed	FL: -0.133<br>HL:0.148	FL: 0.063<br>HL: 0.058	FL: 179<br>HL:179	FL: -2.10<br>HL:2.55	FL: **0.037**<br>HL: **0.012**
		Limb (hindlimb)	Min: 78.5<br>Mean: 56.5<br>Max: 39.1	Min: 12.25<br>Mean: 9.70<br>Max: 10.57	Min: 179<br>Mean:179<br>Max: 179	Min: 6.41<br>Mean: 5.82<br>Max: 3.69	Min: **<0.001**<br>Mean: **<0.001**<br>Max: **<0.001**

Significant interaction *p*-values are in bold.

All variables were rank-transformed prior to analysis. For tests with significant limb-by-speed interactions, individual speed estimates are provided for each limb (FL: forelimb; HL: hindlimb) and intraspecific differences between limb pairs were tested at minimum, mean, and maximum values of the overlapping speed range across limb pairs and species.

Forelimb not listed because it was used as the reference variable.

Journal of Experimental Biology • Supplementary information

Table S4. Post hoc *interspecific* comparisons of single limb impulses following full-factorial analysis of covariance.

Limb	Response Variable	Fixed Effect	Estimate	Standard Error	df	t-value	*p*-value
Fore	Peak Vertical Impulse	Speed	BV: -0.372<br>CD: -0.060	BV: 0.077<br>CD: 0.135	BV: 179<br>CD: 179	BV: -4.8<br>CD: -0.45	BV: **<0.001**<br>CD: 0.656
		Species (*C. didactylus*)	Min: 48.1<br>Mean: 23.7<br>Max: 4.37	Min: 27.2<br>Mean: 24.7<br>Max: 26.2	Min: 5<br>Mean: 5<br>Max: 5	Min: 1.77<br>Mean: 1.02<br>Max: 0.167	Min: 0.135<br>Mean: 0.375<br>Max: 0.874
	Peak Propulsive Impulse	Speed	-0.159	0.0666	180	-2.39	**0.018**
		Species (*C. didactylus*)	11.1	18.1	5	0.61	0.566
	Peak Braking Impulse	Speed	-0.152	0.0675	180	-2.26	**0.025**
		Species (*C. didactylus*)	20.5	17.1	5	1.20	0.285
	Peak Medial Impulse	Speed	-0.344	0.0730	180	-4.71	**<0.001**
		Species (*C. didactylus*)	-21.6	17.1	5	-1.27	0.261
	Peak Lateral Impulse	Speed	-0.344	0.0730	180	-4.71	**<0.001**
		Species (*C. didactylus*)	62.4	11.7	5	5.35	**0.003**
	Peak *Net* Fore-Aft Impulse	Speed	-0.133	0.0634	179	3.34	**0.001**
		Species (*C. didactylus*)	7.13	8.60	5	0.829	0.445
Hind	Peak Vertical Impulse	Velocity	BV: -0.372<br>CD: -0.060	BV: 0.077<br>CD: 0.135	BV: 179<br>CD: 179	BV: -4.8<br>CD: -0.45	BV: **<0.001**<br>CD: 0.656
		Species (*C. didactylus*)	Min: 49.5<br>Mean: 25.1<br>Max: 5.77	Min: 27.8<br>Mean: 25.7<br>Max: 26.2	Min: 5<br>Mean: 5<br>Max: 5	Min: 1.78<br>Mean: 1.02<br>Max: 0.22	Min: 0.137<br>Mean: 0.356<br>Max: 0.835
	Peak Propulsive Impulse	Velocity	-0.159	0.0666	180	-2.39	**0.018**
		Species (*C. didactylus*)	24.2	18.5	5	1.31	0.249
	Peak Braking Impulse	Velocity	-0.152	0.0675	180	-2.26	**0.025**
		Species (*C. didactylus*)	40.0	17.6	5	2.27	0.073
	Peak Medial Impulse	Velocity	-0.344	0.0730	180	-4.71	**<0.001**
		Species (*C. didactylus*)	14.8	17.8	5	0.833	0.443

Journal of Experimental Biology • Supplementary information

	Peak Lateral Impulse	Velocity	-0.344	0.0730	180	-4.71	**<0.001**
		Species (*C. didactylus*)	50.8	12.6	5	4.04	**0.010**
	Peak *Net* Fore-Aft Impulse	Velocity	-0.133	0.0634	179	3.34	**0.001**
		Species (*C. didactylus*)	-29.3	9.13	5	-3.20	**0.024**

Significant interaction *p*-values are in bold.

All variables were rank-transformed prior to analysis. For tests with significant limb-by-speed interactions, individual speed estimates are provided for each species (BV: *Bradypus variegatus*; CD: *Choloepus didactylus*) and inter-limb differences between species were tested at minimum, mean, and maximum values of the overlapping speed range across limb pairs and species.

B. variegatus is not listed because it was used as the reference variable.

Table S5. Subset of means (±s.d.) for impulses (normalized in %BWS) for *Bradypus variegatus*.

Speed (ms^{-1})	Limb	*N*	Contact Time (s)	J_V (%BWS)	J_P (%BWS)	J_B (%BWS)	J_M (%BWS)	J_L (%BWS)
0.08±0.02	FL	20	4.67±2.09<br>(4.20-5.14)	166±90.4<br>(146-186)	24.4±15.0<br>(21.1-27.8)	–6.2±16.7<br>(–10.5- –1.9)	–8.5±12.4<br>(–11.5- –5.5)	12.5±14.9<br>(8.9-16.1)
	HL	20	5.07±2.80<br>(4.44-5.69)	207±121<br>(180-234)	6.0±12.6<br>(2.9-9.1)	–42.0±30.9<br>(–49.0- –35.1)	–16.3±15.5<br>(–20.1- –12.4)	14.7±25.1<br>(8.6-20.8)

N, number of trials for which consecutive fore- and hindlimb forces were collected; FL, forelimb; HL, hindlimb; BWS, bodyweight seconds; J_V, vertical impulse; J_P, propulsive impulse; J_B, braking impulse; J_M, medial impulse; J_L, lateral impulse.

In parentheses are 95% confidence intervals.

Vertical impulse applied by the animals is shown as positive (absolute) values by convention, as well as (+) propulsive force and (-) braking force.
